# Supplementary material for: Are carbon emissions trading and green financial instruments synergistic? -Comprehensive quantitative research based on content analysis
Source: PLoS One. 2024 Mar 7;19(3):e0298601. doi: 10.1371/journal.pone.0298601 (PMC10919601; doi:10.1371/journal.pone.0298601)
Supplement: S1 Data — (ZIP) [file pone.0298601.s001.zip › Data/地级市Z_F工作报告.zip!/╔╜╢1⁄2/╠⌐░▓╩╨/└┤╫╘╣1⁄2╓┌║┼í╛┬φ┐╦╩2╛▌═°í┐_2010.docx]

各位代表：

　 现在，我代表市人民政府向大会报告政府工作，请予审议，并请市政协委员和其他列席人员提出意见。

　 一、过去的一年，全市经济社会实现了平稳较快发展

　 2009年是新世纪以来我市经济社会发展最为困难的一年。面对国际金融危机的冲击和各种挑战，全市人民在中共泰安市委的领导下，以党的十七届三中、四中全会精神为指导，深入贯彻落实科学发展观，认真落实中央和省扩内需、保增长、调结构、惠民生的一系列政策措施，坚定信心，共克时艰，科学务实，积极作为，全市经济社会实现平稳较快发展，圆满完成了年初确定的目标任务。

　 (一)经济运行质量较好，居民收入增长较快。预计全市实现生产总值1700亿元左右，比上年增长13%左右。完成地方财政收入91.4亿元，增长19.7%，其中各项税收占财政收入的比重达到79.9%，提高3.2个百分点。规模以上固定资产投资完成1031.9亿元，增长24.9%。实现社会消费品零售总额555亿元，增长19%左右。金融机构人民币各项存、贷款余额分别达到1213.2亿元和769.6亿元，比年初增长24.5%和27.2%。城镇居民人均可支配收入达到17700元、农民人均纯收入达到6620元，分别增长10%左右和9%左右。

　 (二)调结构、转方式取得积极进展。农业和农村经济发展势头良好。全年粮食总产创历史最好水平；新增高效经济作物15万亩、有机食品认证5万亩、有机蔬菜3.6万亩，苗木花卉达到26.7万亩，奶牛存栏达到18.8万头，肉蛋奶总产量达到98.3万吨；新上规模以上农业龙头企业104家。工业经济持续回升向好。规模以上工业实现增加值934.2亿元，增长15.6%；实现主营业务收入2980亿元、利税370亿元、利润220亿元，分别增长17%左右、17%以上、17%以上；主营业务收入过亿元、10亿元的工业企业分别达到565家、45家，比上年增加125家、10家，百亿企业达到3家；利税过千万元、亿元的企业分别达到608家、55家，增加105家、5家。旅游服务业快速发展。国内旅游人数达到2321万人次，实现国内旅游收入177.8亿元，分别增长25.6%和29.9%，泰山被列为国家5A级旅游景区免检单位；服务业增加值增速超过GDP增速2个百分点，占GDP的比重提高1个百分点。自主创新、节能减排、矿产资源整合开发和生态市建设取得新进展。53项成果获国家、省科技进步奖励，按国家新标准认定高新技术企业15家，累计达到35家，新增省级名牌14个，中国弛名商标达到10个；泰安再次荣获“全国科技进步先进市”称号，被列为国家知识产权示范创建市。万元GDP能耗、规模以上工业万元增加值能耗、二氧化硫和COD排放量持续下降。

　 (三)大项目建设、招商引资实现新突破。全市确定的125个重点建设项目完成投资449亿元，占年度计划投资的116.6%。全市共争取新增中央投资项目4批、229个，下达中央资金4.1亿元；争取省调控资金项目53个、11.0亿元。全市实际利用外资11.8亿美元，增长0.5%；3000万元以上国内招商引资项目实际到位资金229亿元，增长10.6%。莲花汽车、泰山方特欢乐世界、泰山封禅大典大型实景演出、泰开电气双百万变压器和超高压交联电缆、岱岳区科诺型钢、新泰华溢碳纤维、肥城石墨电极、宁阳合浩通电缆、东平宏达矿业等项目顺利建设或投产，提升了产业层次，增强了发展后劲。

　 (四)创建国际旅游名城工作扎实推进。按照“一年一大步、三年大变样”的目标要求，规划了十大重点工程、105个建设项目，总投资321亿元，目前已开工97个，竣工35个，环山带工程、文化产业项目、大汶河综合开发、城中村改造及旅游经济开发区、高铁车站新片区基础设施建设加快推进。统筹城乡发展，集约节约用地，编制了《泰安市土地利用总体规划》、《泰安市城市规划区城乡统筹总体规划》和城市重点工程、重点地段的设计或详细规划。泰城实施了环境综合整治“四大工程”、“四大专项行动”，城区环境面貌和交通经营秩序有了明显改观。县市驻地和小城镇规划、建设与管理取得新进展。

　 (五)民生改善、社会建设取得显著成绩。全市新增城镇就业再就业6.8万人，农村劳动力转移就业7.2万人，城镇登记失业率3.26%；收缴各项社会保险费48.4亿元，增长22.1%。城乡义务教育经费保障机制逐步完善，为51.6万名城乡义务教育段学生免除学杂费，全市实现了县域内教师工资统筹发放。公共文化服务体系覆盖面进一步扩大，广播电视村村通、“农家书屋”等文化惠民工程积极推进。城乡医疗卫生基础设施建设和服务能力明显增强，新型农村合作医疗参合率达到98.9%，城市社区卫生服务覆盖率达到100%，继续保持全省先进水平。人口计生工作连续19年保持低生育水平，全市合法生育率保持在97%以上，荣获全国首批人口计生综合改革示范市称号。城镇居民基本医疗保险制度全面建立，困难群众救助、城乡低保、五保供养等工作都有新的进展。市委、市政府确定为民要办的12件实事全面完成。全市新建廉租住房10.7万平方米、经济适用房5.1万平方米，其中市区新建廉租住房4.5万平方米，经济适用住房2.8万平方米，对符合廉租住房条件并提出申请的家庭做到了应保尽保。启动了农村住房建设与危房改造，全市已有245个村实施了整村迁建，改造危房8967户。累计归集住房公积金39.8亿元，为3.4万名职工发放贷款32.2亿元。完成143座小型水库除险加固，大中型水库除险加固完成年度任务。

　 全市行政村通油路率、通客车率达到97.6%、99.5%。完成农村无害化生态卫生厕所5.7万座，新建户用沼气池1万个。全运圣火泰山采集、泰安传递和赛事承办圆满成功，全民健身活动和体育场馆建设取得新成绩。“平安泰安”建设深入开展，治安防控体系不断完善，打击刑事犯罪力度不断加大，安全生产、应急管理和食品药品监管得到加强。庆祝建国六十周年各类活动丰富多彩，精神文明建设成效明显。援建北川、美国白蛾防治、重大动物疫病防控、甲型H1N1流感和手足口病疫情防控等项工作取得阶段性成果。民主法制、国防动员、人民防空、民兵预备役、双拥共建取得新成绩。妇女儿童和残疾人保障、老龄工作及服务有新的提高，防震减灾三大体系建设取得新进展，新编《泰安市志》出版发行，外事侨务、民族宗教、气象、档案等项社会事业全面进步。

　 (六)政务服务能力和水平有新的提高。全面清理规范性文件及其他政策性文件，废止144件，修改24件，进一步规范了行政行为。积极开展行政效能监察和民主评议政风行风活动，完善涉企检查、收费备案和首次不罚制度，创新执法监督方式，集中开展专项治理，发展环境不断优化。深入实施《公务员法》，大力加强公务员队伍建设，干部队伍素质和施政能力不断提高。加快推进网络审批平台建设，积极推行并联审批和标准化服务，审批服务质量和效率进一步提高。政务信息化、政务公开、便民服务等深入开展。按照上级部署，研究制定了市政府机构改革方案。投融资管理、文化市场管理体制等进一步理顺，政府服务效率和质量有了新的提高。

　 各位代表，在世界经济深度衰退、国内外形势严峻复杂的情况下，全市经济社会实现平稳较快发展实属不易。这是我们在市委的坚强领导下，认真贯彻中央和省决策部署、全面落实科学发展观的结果；是全市上下同心同德、艰苦奋斗的结果；也是驻泰各单位和各界人士鼎力支持、自觉融入泰安经济与社会发展的结果。在此，我代表泰安市人民政府，向全市广大干部群众、各民主党派、工商联、无党派爱国人士，向人民解放军和武警驻泰部队及省以上驻泰单位，向关心支持泰安发展的港澳台同胞、海外侨胞及海内外朋友，表示崇高的敬意和衷心的感谢！

　 在充分肯定成绩的同时，我们也清醒地看到面临的严峻形势和发展中存在的困难和问题。国际金融领域风险尚未消除，全球经济复苏可能是缓慢复杂曲折的过程。国内经济回升向好的基础虽然逐步稳固，但经济社会发展仍面临较大的不确定性，制约经济回升的因素还比较多。我市经济结构不够合理、发展方式粗放，传统产业比重大，高新技术产业比较薄弱，服务业发展较为滞后；骨干企业总量少、个头小，企业自主创新能力不强；经济增长过多地依赖投资，消费拉动能力不强，外向型经济尤其是出口总量小，经济发展的活力不足；城镇化水平低，城乡之间、经济与社会发展之间不平衡；财政增支减收因素明显增多，收支平衡压力加大；农民增收缓慢，劳动就业、社会保障等改善民生的任务还很繁重；推动科学发展的能力还需进一步提高，转变政府职能、提高行政效能和服务质量、优化发展环境、加强廉政建设等还有许多工作要做，等等。对此，我们将采取更加有力的措施，认真加以解决。

　 二、审时度势，科学谋划好2010年的工作

　 2010年是实施“十一五”规划的最后一年，也是有效应对国际金融危机的关键之年。做好今年的工作，对于巩固经济回升向好的势头、为“十二五”规划启动实施奠定良好基础具有十分重要的意义。政府工作的总体要求是：认真贯彻党的十七大和十七届三中、四中全会以及中央、全省经济工作会议精神，坚持以邓小平理论和“三个代表”重要思想为指导，深入贯彻落实科学发展观，按照市委确定的“一三四五”发展思路，紧紧围绕加快经济发展方式转变战略任务，着力调整经济结构，着力抓好项目建设，着力推进城镇化和“创城”工作，着力推进改革开放创新，着力保障和改善民生，继续实施事关全局的重点带动战略，提高经济增长质量和效益，推进经济平稳较快发展，促进社会全面进步，全面完成“十一五”规划目标任务，推进建设经济文化强市、打造国际旅游名城取得新进展，为实施“十二五”规划打下坚实基础。主要预期目标和约束性指标是：全市生产总值增长13%左右；地方财政收入增长14%；全社会固定资产投资增长23%左右；社会消费品零售额增长17%以上；外贸进出口和实际利用外资实现恢复性增长；城镇登记失业率控制在3.8%以内；人口自然增长率控制在8.1‰以内；城镇居民人均可支配收入和农民人均纯收入均增长8%以上；万元GDP能耗、规模以上工业万元增加值能耗、二氧化硫和COD排放量全面完成“十一五”规划目标。在实际工作中力求发展得更好一些、更快一些。

　 做好今年的工作，挑战和机遇并存，机遇大于挑战。从国际看，在各国政府经济刺激政策的作用下，世界经济复苏可能早于预期，有望实现恢复性增长，世界经济环境将好于去年。从国内看，经济回升向好的基础逐步巩固，发展的活力与动力将进一步增强；国家继续实施积极的财政政策和适度宽松的货币政策，将极大地提高经济发展的可持续性、均衡性、协调性，这是我们实现平稳较快发展的宏观保障。从我市看，坚持不懈地抓招商引资和项目建设，发展的后劲和支撑作用明显增强，尤其是通过连续两年开展深入学习实践科学发展观教育活动，全市上下求发展、抓发展的积极性进一步高涨。只要我们审时度势，坚定信心，抢抓机遇，积极作为，全市经济与社会就一定能够实现平稳较快发展。在工作指导和把握上，要做到“六个必须”：一是必须始终坚持以科学发展观统揽全局，紧紧扭住发展第一要务不放松，处理好保增长与促转变的关系，处理好消费、投资、出口拉动增长的关系，统筹推进经济与社会全面协调可持续发展。二是必须把上级指示精神与泰安实际相结合，创造性地开展工作，紧紧抓住项目建设和招商引资不放松，加大“创城”工作力度，全面提升城镇化水平，努力打造经济社会发展的新优势。三是必须坚持调结构、转方式，毫不放松地抓好经济结构调整、自主创新、节能减排和节约集约用地，在提升经济发展质量、效益和持续发展的后劲、竞争力上狠下功夫。四是必须高度重视体制机制创新，认真研究解决发展中的瓶颈制约问题，深化改革，扩大开放，增强经济发展的动力和活力。五是必须始终坚持以人为本，更加注重改善民生，认真解决好人民群众最关心最直接最现实的利益问题，切实维护社会和谐稳定。六是必须坚持继续解放思想，切实增强危机感、紧迫感、责任感，积极作为，科学务实，调动各方面的积极性，合力开创各项工作新局面。

　 三、着力调结构、转方式，构筑经济发展的新优势

　 调结构、转方式是深入贯彻落实科学发展观的重要目标和战略举措，也是今年经济工作的重大任务和主攻方向。必须坚持在调整中促转变、在转变中促发展，加快经济转型升级步伐，坚决打好转方式、调结构这场硬仗。

　 (一)加大农业结构调整力度，促进农业增效和农民增收。按照稳粮保供给、增收惠民生、改革促统筹、强基增后劲的要求，夯实“三农”工作基础。一是稳定粮食生产。认真组织实施国家新增千亿斤粮食产能规划，继续推进土地开发整理复垦和基本农田示范化建设，加大中低产田改造、高产示范片创建和二期大型商品粮基地建设。二是大力推进农业产业化。重点培植一批产业关联度高、带动能力强、有较强竞争力的大中型农产品加工企业，大力发展农民专业合作社，拉长展宽农业增效、农民增收的链条。年内新发展规模以上龙头企业35家，农民专业合作社200家以上。继续抓好“一村一品、百村示范、千村推进工程”和“奶业富民”工程，着力发展有机蔬菜、干鲜果品、苗木花卉等优质、高效、特色农产品。年内新增有机食品认证面积5万亩，其中有机蔬菜3万亩，奶牛养殖达到20万头，苗木花卉达到28.2万亩。加快农产品质量检测体系和出口标准化基地建设，全面提升质量监管能力和水平，确保农产品安全。三是加强农业生产能力建设。切实抓好以水利为重点的农田基本建设，全面完成12座大中型、130座小型水库除险加固，搞好水土流失综合治理、节水灌溉、造林绿化、农业综合开发。加快推进农村电网改造、饮水安全和农村道路等建设，提高农机技术和装备水平，加快建立多元化的农业科技推广体系，不断改善和提升农业生产条件。加强重大动物疫病防控工作。四是多渠道增加农民收入。加快农村二、三产业发展，加强农民工技能培训、就业指导服务和权益保护，抓好农村劳务输出。年内全市新增农村劳动力转移就业6万人。全面落实好粮食直补、良种补贴等强农惠农政策。积极探索土地承包经营权流转的有效形式，深化农村集体林权、水利、金融和综合配套等项改革。

　 (二)加快工业结构调整，提升工业竞争力。年内全市新增规模以上企业200家，规模以上工业增加值增长14%以上。一是大力发展高端高质高效产业和战略性新兴产业。研究制定和实施我市高端产业发展规划，大力发展高新技术产业，积极运用高新技术改造提升传统产业，加快发展高端旅游、文化和现代服务业，引导高端产业集中布局，集约发展，努力打造我市高端产业聚集区。大力培植战略性新兴产业，加快发展新能源、新材料、新医药、新信息和特色优势产业。根据不同产业特点，制定发展规划和实施方案，确定发展重点和市场推进措施，集中力量开发一批新产品，上一批新项目，形成一批新产业。注重发挥好高新技术产业开发区的载体作用，引导战略性新兴产业向园区聚集，提高新兴产业发展的集聚度和带动力。二是抓好主导产业和骨干企业培植。认真落实工业结构调整振兴意见和汽车、煤炭、输变电设备等八大主导产业振兴规划，围绕规划上项目、抓招商，打造产业“网链”，力求形成核心企业带动、中小企业集群发展的局面。积极推进信息化与工业化融合，加快传统产业改造升级，依靠高新技术嫁接改造，提高传统产业的技术含量，增强创新能力，提高装备水平，多出高端产品，拉长产业链条。围绕主导产业的培植，引导骨干企业实施“双百”工程，既要抓投入膨胀规模，又要抓管理提升素质，打造一批百亿企业和百年企业。三是加快发展中小企业。认真落实国家、省扶持中小企业发展的若干政策措施，抓好科技创新创业、中小企业成长、特色产业提升和小企业培育四个计划的实施，加强金融、信息、科技、担保、法律服务体系建设，进一步解决中小企业融资难、人才技术引进难等问题，培育一大批成长性强、对财税贡献大的中小企业和特色产业集群。

　 (三)加快服务业结构调整，推动服务业发展上水平。一是积极壮大培育旅游支柱产业。以建设山东旅游和文化产业高地为目标，发挥泰山旅游龙头带动作用，坚持拓展旅游空间、建设旅游项目、开发旅游产品、完善旅游设施、提升服务水平、加强宣传促销多管齐下，积极推进大泰山旅游圈的规划建设，努力做好“吸引人、留住人”的文章。加快推进“数字化泰山”进程，抓好泰山瑞奥不夜城、盐海神汤、大泰山生命颐养度假区、地下大裂谷、桃花峪游人中心、泰山汉御道等重点旅游项目和重大旅游设施建设，加快旅游经济开发区、徂徕山、莲花山、水浒文化旅游区等资源开发，抓好星级酒店、特色餐饮美食街、旅游购物休闲街、旅游集散中心建设，完善景区和泰城旅游标识，提高旅游各要素的服务水平。借助“好客山东贺年会”品牌，开发具有地方特色的活动和产品，树立“新泰安、新体验”旅游形象，不断增强对游客的吸引力。开展“旅游服务质量提升年”活动，加强旅游市场主体建设，积极引进、培强、做大一批旅游骨干企业。二是高度重视现代服务业发展。制定实施现代物流业振兴规划，加快推进服务业“103030”工程载体建设，重点抓好金融保险、现代物流、信息服务、创意研发等生产性服务业，提高生产性服务业对制造业的支撑能力。积极发展生活性服务业，重点抓好体育健身、医疗保健、职业培训、老年护理、家政服务等具有巨大潜在需求和发展空间的社会服务业，满足居民多元化需求。三是大力培育文化产业。以打造泰山文化、大汶口文化为重点，以园区、项目建设为支撑，推进文化体制改革，培育文化市场主体，发挥文化产业发展专项资金的作用，加强旅游与文化的融合，大力发展文化旅游业、文化演艺业、节庆会展业、现代传媒业。积极发展以数字、网络等高新技术为支撑的新兴文化业态，抓好文化创意、出版发行等产业发展，培育一批文化创意群体，提高文化产业对经济增长的贡献率。四是增强消费对经济增长的拉动作用。大力启动农村消费，落实好一系列开拓农村市场、扩大农村消费的政策措施，抓好家电、汽车、摩托车下乡和以旧换新工作。积极培植城市消费热点，推动消费结构优化升级。加强和改善对房地产市场的调控，增加有效供给，促进房地产业健康发展。

　 (四)着力提升自主创新能力，大力推进节能减排。坚持把自主创新作为转方式、调结构的中心环节，大力发挥科技、人才、管理等生产要素作用，努力促进经济增长由主要依靠增加物质消耗向主要依靠科技进步、劳动者素质提高、管理创新转变。一是强化创新体系建设。充分发挥政府的主导作用、企业的主体作用、科研机构和大专院校的引领作用，真正建立起产学研相结合、协同推进的科技创新体系。强化科技投入机制建设，形成政府、企业、社会三方结合、灵活多样的创新投入方式，有效整合各类科技资源，加大创新力度，增强创新能力。二是强化创新型企业培植。紧紧围绕我市主导产业和骨干企业的培植，选择一批重点培育的骨干企业，突出重点领域和关键技术，持续不断地加大科技投入、科技研发、科技成果转化力度，使其真正引领和带动全市科技创新。支持企业技术中心、科技孵化中心、大学科技园、科技创业园等创新服务平台和各类科技中介服务机构建设，引导创新要素向企业集聚，促进科技成果向现实生产力转化。确保全市高新技术产业产值占规模以上工业总产值的比重提高2个百分点。加大企业创新项目建设力度，积极争取国家、省重要科技创新平台、重大科技项目、科技创新资金落户泰安。三是强化创新人才支撑。大力实施科教兴市和人才强市战略，加强各类人才队伍建设，尤其要紧紧围绕产业发展和科技创新，加快培育、引进高科技人才、专业技术人才、高技能人才。大力加强企业家队伍建设，完善企业家培训培育和激励约束机制，加快企业家市场化、职业化步伐，建设一支有远见、善创新、懂管理、会经营的战略性企业家队伍。尊重人才、尊重创造、尊重创业，努力创造人才辈出、人尽其才的良好环境。

　 节能减排是调结构、转方式的必然要求和成果体现。今年是完成“十一五”节能减排目标的决战一年，必须毫不松懈地确保完成。一是坚持两手并重。即一手抓淘汰落后，把任务目标分解到县、乡，把责任落实到单位，加大督促考核，确保全面完成“十一五”规划确定的淘汰水泥357.4万吨、关停小火电25万千瓦等落后产能目标任务；一手抓源头控制，采取严格市场准入、强化环境监管、依法供地用地、严格项目审批等措施，防止“两高”行业低水平重复建设。继续推进10大重点节能工程、重点流域环境治理、燃煤电厂脱硫治理，突出抓好市百户重点用能企业和50个重点节能项目。大力推广使用高效节能技术和产品，加快推进太阳能与建筑一体化。二是大力发展循环经济。深入实施“1623培育计划”，重点抓好100个循环经济项目，培育6个循环经济型园区、20家清洁生产示范企业和30家循环经济型企业。三是加强生态环境建设。认真实施《泰安生态市建设总体规划》，以水、大气、土壤等污染防治为重点，加快治污项目建设；以绿化、美化等为重点，加强城乡人居环境建设。加大检查监督力度，健全完善环境资源补偿机制、节能环保工作机制、统计体系和考核奖惩制度。大力宣传节能环保知识，树立全民节能环保意识，在全社会形成节能环保的浓厚氛围。

　 四、加大项目建设、招商引资力度，增强可持续发展的后劲和活力

　 项目建设、招商引资是扩内需、稳增长、调结构、转方式的根本途径，是巩固推进经济企稳向好势头的重要支撑，必须坚持不懈地抓在手上、抓出成效。

　 (一)坚持不懈地抓好项目建设。一是加快在建和新上项目建设。对94个已批未完工新增中央投资、省调控资金项目，按照项目开工率、资金配套率和问题整改率“三个百分之百”的要求，集中力量抓好建设，尽快形成更多的实物量。突出抓好重汽工业园、泰开工业园、现代重工装载机、新凯汽车、泰山石膏股份公司石膏板护面纸、澳门名嘉城市广场、华能风力发电、特变电工工业园、石横特钢公司高强度钢及特种钢改造、合浩通180万千米电缆、中顺工业园等100个左右投资过亿元的重点项目建设，力争早投产、早见效。积极支持京沪高铁、东平铁路、中石油天然气管网泰安段等重大基础设施项目建设，力争济泰城际轨道交通、青兰高速泰聊段、104国道泰城段改线等项目早日开工，抓好济徐高速东平段和京杭运河济宁—东平段复航等工程建设。二是突出抓好项目的筛选储备。围绕交通、能源、农林水利、社会民生、高新技术、研发改造、现代服务业、住宅和房地产开发等八大重点领域，采取政府推动、企业策划、委托策划等多种形式，策划论证一批符合国家产业政策、对结构调整和产业升级有引领作用的大项目、好项目。年内完成80个重点项目的策划任务。加大对上争取力度，用足用好用活国家和省里的各项政策，力争有更多的项目、更多的资金落户泰安。三是创新项目建设体制机制。创新项目建设服务机制，认真落实领导干部包重点项目责任制和定期走访客商、企业制度，探索成立投资服务中心，具体负责为全市重点项目代理报批手续，协调落实优惠政策，帮助解决建设与经营中遇到的困难和问题。创新项目融资机制，采取银行贷款、招商引资、企业自筹、民间投资、上市融资等多渠道融通项目建设资金，保持投资合理增长。落实民间投资各项扶持政策，放宽市场准入范围，完善民间投资担保机制，把民间投资的巨大潜能激发出来。创新项目建设推进机制，强化责任落实，强化项目调度，强化工作考核，加快项目建设进度。

　 (二)千方百计扩大招商引资。一是创新招商体制机制和方式方法。以产业化招商为主导，加快健全完善领导分管、部门负责、专业运作、企业(园区)承载“四位一体”的产业招商工作体制。进一步完善招商引资工作的考核机制、激励机制和重点招商项目推进机制，调动各级干部和群众招商引资、干事创业的积极性。更加注重招商引资实效，进一步创新委托招商、以商招商、园区招商、网上招商、会展招商等方式方法。坚持跟踪抓好在谈、在批、在建项目，办好重大招商活动，加快招商项目晋位升级。加大政府招商力度，把“招大引强”、“招新引优”、“招才引智”摆上更加突出的位置。二是强化企业招商的主体作用。鼓励企业适应新的全球产业转移、资本流动趋势，有效利用国内外强企业的跨国并购、增资扩股、股权投资、资产重组、经营合作等多种方式，扩大引进资金规模。强化大企业、大项目招商，搞好招商项目的策划推介，选准国内外有实力的大企业、大集团尤其是“央字号”大国企，组织专门力量，有针对性地开展招商引资，力争在引进战略投资者和具有重大带动作用的大项目上实现新突破。三是扎实做好外经贸工作。坚持与壮大主导产业相结合、与企业技术改造相结合、与龙头企业扩规扩能相结合、与培育境外上市资源相结合，策划引进一批能够拉动进出口、拉动加工贸易的外资项目，实现利用外资和出口总量的双推进。实施市场多元化战略，加快出口企业基地建设，调整出口商品结构，培育出口名牌企业，积极开拓新的市场，努力保市场份额，提升企业国际竞争力。实施“走出去”战略，鼓励有条件、有实力的企业有序向外转移生产能力、开展境外资源合作开发和承包境外工程。四是进一步优化提升招商引资环境。加强园区和城镇基础设施建设，完善各项服务功能，优化人居创业环境，增强对外商投资的承载力和吸引力；讲信用、重承诺，多服务、少检查，多帮忙、不添乱，尽心竭力为外商和企业的发展创造良好环境和条件，努力营造亲商、安商、富商的浓厚氛围。

　 (三)充分发挥财税金融对经济社会发展的支持和引导作用。努力保持财税收入稳定增长，培植壮大优质高效财源税源，优化财税收入结构，改善收入质量。立足调结构、转方式，优化支出结构，进一步加大对科技创新、节能减排、现代农业、服务业、文化产业的财政支持力度，加大教育、就业、社会保障、医疗卫生等方面的投入。深化国有企业改革，完善国有资本经营预算制度，提高国有和国有控股企业税后利润上缴财政比例，规范高管人员薪酬和国有企业投资行为。继续完善廉租房补贴政策，加大廉租住房建设和农村危房改造的支持力度，落实好鼓励住房消费的各项财税政策。牢固树立现代财税理念，加强财政投资评审管理，严格财税监管，提升财税管理的精细化、科学化水平。继续优化金融生态环境，加强信用体系建设。大力支持金融业改革发展，积极发展小额信贷组织、村镇银行、贷款公司、农村资金互助社等新型农村金融机构。各金融机构要积极落实适度宽松的货币政策，满足企业发展的资金需求，增强金融对经济发展的支持力度。搞好上市资源培育，引导支持企业上市融资，尤其是鼓励企业积极参与国际化竞争，实现境外上市融资。

　 五、以“创城”为抓手，加快推进城镇化进程

　 加快推进城镇化，是推动经济与社会长期稳定持续增长的重要力量。按照统筹城乡、合理布局、节约土地、完善功能、以大带小的原则，创新理念，加大工作力度，加快推进城镇化进程，走资源节约、环境友好、设施配套、经济高效、文化繁荣、社会和谐、城乡共荣的新型城镇化道路。

　 (一)强化规划的引领和刚性作用。认真实施泰安市土地利用规划、城市总体规划和历史文化名城保护规划，科学编制到2030年的《泰安市城镇规划体系》。城镇规划要突出科学性、前瞻性、特色性和协调性、可持续性，注重产业带动、环保节能、科技引擎、功能完善配套和品位档次的提升。切实抓好城市控制性详细规划和村镇建设规划，推进城乡规划全覆盖，为高标准建设提供科学依据。强化规划的刚性作用，严格按规划建设管理，确保规划的严肃性。

　 (二)构筑具有泰安特色、功能相互衔接的城镇体系。按照泰安中心城市、县域城市、小城镇、农村(社区)四个层面，合理布局，搞好开发建设。做强做大泰城，增强对全市的辐射带动作用。按照“一年一大步，三年大变样”目标，继续大力推进创城重点工程建设，增强品位、功能和吸引力。坚持东西两翼拓展、南部新城开发、旧城改造提升，着重抓好高新区、旅游经济开发区、高铁车站新片区、东部旅游产业聚集区的综合开发和城中村改造，集中力量抓好会展中心等重点项目建设。借助大汶河综合开发，逐步实现泰城由“依山而建”向“依山傍水”发展。实施“两轴”、“两带”带动战略，加快老城区改造与新城区开发步伐。加快膨胀提升县城和小城镇，增强对农村的辐射和带动作用。突出产业经济、基础设施、公共项目建设，扩胀规模，提升质量。实施扩权强镇，集中力量抓好4个国家重点镇和15个省级中心镇建设。改革户籍管理制度，制定并落实鼓励政策，吸引农民进城进镇创业经营。以中心村为核心，科学规划，有效整合农村资源，切实抓好社会主义新农村社区建设，发展社区服务，努力提升农村居民生活质量和水平。

　 (三)创新城镇发展模式。创新投融资机制，深化完善政府主导、市场化运作有机结合的运行机制，吸引更多的资金参与城镇化建设。强化集约发展、片区开发理念，整合优化城市资源，突出抓好城中村改造、农村住房建设与危房改造，加快重点片区开发。高度重视节地、节能、节水、节材和资源综合利用，更多地依靠制度创新、科技创新、内涵式增长推动节能减排，发展循环经济，建设低碳城市，促进资源节约型、环境友好型社会和生态文明建设。创新管理体制，坚持综合整治与建立长效管理机制相结合，广泛动员社会力量，搞好城市净化、绿化、亮化和美化，真正做到人民城市人民建、人民城市人民管。

　 六、高度关注和改善民生，统筹促进社会事业发展

　 改善民生既是发展的重要目的，也是发展的动力源泉，是拉动最终消费最有效的手段。必须从“扩内需、调结构、促发展”的战略高度，千方百计改善民生，促进社会事业健康发展。

　 (一)积极做好就业和社会保障工作。把促进就业作为经济社会发展的优先目标，继续实施更加积极的就业政策，以创建“国家级创业型城市”为契机，大力实施全民创业战略，加强对全民创业的政策服务、培训指导、资金支持，搞好“百千万”工程，以创业带就业。切实做好高校毕业生、农民工、复退军人和下岗失业人员就业再就业。积极开展就业援助，开发更多社会公益岗位，帮助“4050”人员、“零就业家庭”、残疾人、低保对象、被征地农民等困难人员就业。年内全市新增城镇就业再就业5.8万人。坚持落实“保发、促缴、扩面、严管”方针，增加公共财政投入，不断扩大养老、医疗、失业等各项社会保险覆盖范围，着力抓好非公有制经济从业人员、农民工和灵活就业人员的参保工作，加快建立覆盖城乡居民的社会保障体系，不断提高社会保障水平。进一步完善企业职工基本养老保险市级统筹，扎实做好包括农民工在内的参加城镇企业职工基本养老保险关系跨省市的转移接续工作。把农村社会保障制度建设摆在突出位置，稳妥推进国家首批新型农村社会养老保险试点。健全完善企业工资正常增长机制，落实好国家提高企业退休人员基本养老金、部分优抚对象待遇政策。进一步提高城乡低保水平，农村低保标准提高到每人每年不低于1200元，城市低保对象月人均补助水平提高到不低于160元，实现新标准下的应保尽保。实施贫困残疾儿童康复救助计划，加大残疾人生活救助力度，重度残疾人每人每月增加50元生活补贴。大力发展社会福利、社会救助和慈善事业，搞好社会福利服务中心建设。加快推进保障性安居工程、农村住房建设和危房改造工作，年内完成惠普家园南区一、二期工程，新建农村住房6万户、改造危房1.5万户。进一步提高住房公积金制度覆盖面，扩大贷款规模，强化资金监管，确保资金安全。集中财力，重点办好一批经济发展急需、人民群众热切期盼的实事。

　 (二)繁荣发展教育卫生文化等社会事业。坚持教育优先发展战略，健全教育投入保障机制，推进优质教育资源共享，促进城乡区域教育均衡发展；完善助学政策，保障困难家庭和农民工子女接受义务教育的权利；加大素质教育推进力度，完善教育考试与评价机制；加强中小学办学条件标准化建设，大力实施中小学校舍安全、农村中小学教学仪器更新和“两热一暖一改”工程建设，积极改善办学条件；大力发展职业成人教育特别是农村职业教育，培育一大批实用型技能人才；继续支持高等教育的改革和发展，重视利用好驻泰的高校人才、技术等资源优势。积极推进医药卫生体制和基本医疗保障制度改革，今年城镇居民医保财政补助标准不低于100元，新农合人均筹资标准提高到120元，在政府办基层医疗卫生机构逐步推行基本药物制度；加强城乡基层医疗卫生设施建设，进一步提升村级卫生室和社区卫生服务中心水平；加强重大疫情防控，提高公共卫生事件应急处置能力。健全完善市、县、乡、村四级公共文化服务网络，继续实施广播电视村村通、文化信息资源共享、乡镇综合文化站、农村电影放映、农家书屋建设等文化惠民工程。继续强化人口和计划生育目标责任制，进一步稳定低生育水平。大力开展爱国卫生运动。深入贯彻落实《全民健身条例》，抓好体育场馆建设，努力提高竞技体育水平。加强民主法制建设，大力推进群众性精神文明创建活动。认真落实党的民族政策，加强民族团结，大力发展少数民族经济，依法加强宗教事务管理。重视抓好国防动员、民兵预备役和新一届国家、省双拥模范城(县)创建工作。积极应对老龄化形势，全面推进老龄工作。关心支持妇女儿童、残疾人事业，做好外事、侨务和史志、档案、气象、防震减灾等项工作。

　 (三)努力保持社会和谐稳定。加大“平安泰安”建设力度。强化发展第一要务、稳定第一责任意识，完善社会治安综合治理防控体系，严厉打击各类违法犯罪活动。深入开展“安全生产基层基础年”活动，做到安全警钟长鸣、安全隐患常除、安全事故严查，加强重点行业、重点企业专项整治和安全管理，杜绝重特大安全事故发生。重视抓好食品药品质量安全，严厉打击食品药品制假售假行为。加强社会管理，健全完善社会稳定预警、应急管理和矛盾纠纷化解机制，开展法律援助活动，积极预防和妥善处置各类群体性、突发性公共事件。高度重视人民群众来信来访，妥善处理土地征用、拆迁安置、企业改制、涉法涉诉等方面的矛盾和问题，维护社会和谐稳定。

　 七、加快行政管理体制改革，建设服务型政府

　 新形势、新任务对政府工作提出了新的更高要求。各级政府必须按照创新、务实、高效、清廉的要求，努力加强自身建设，增强应对复杂局面和推动科学发展的能力。

　 要加快政府职能转变。积极稳妥地推进市、县政府机构改革，加快政府职能转变。深化行政审批改革，进一步清理和规范行政审批事项，确保政府机构改革中要求取消、下放、转移的行政审批和管理事项落实到位，真正做到有所为、有所不为。理顺政府公共资源管理体制，建立公共资源交易平台，更大程度地发挥市场在资源配置中的基础性作用。着力推进政企分开、政资分开、政事分开、政府与中介组织分开，把社会管理和公共服务放在更加突出的位置，增强政府提供公共服务的能力。加快推进行政问责制度化、规范化进程，加大对行政不作为和乱作为的问责力度，切实做到有权必有责、用权受监督、违法要追究。

　 要着力推进政务公开。加大政府信息公开力度，围绕社会广泛关注、事关群众切身利益的重大事项，采取多种形式，及时准确地向社会、群众予以公开，保障公众知情权。强化政府决策公开，凡涉及群众切身利益的重大决策都要引导群众有序参与，听取群众意见，接受群众监督。坚持向人大报告工作和向政协通报工作制度，自觉接受人大的法律监督、政协的民主监督和社会舆论监督。优化权力运行流程，加强权力运行监督，切实做到权力在阳光下运行。深入推进办事公开，进一步提升行政审批、便民服务电话受理等政务服务中心功能，更好地方便群众办事。加快推进依法行政，严格按照法定权限和程序行使权力，履行职责，规范行政行为，切实保护公民法人的合法财产和正当权益。

　 要不断提高施政能力。按照建设学习型政府的要求，把学习摆在更加重要的位置，不断加强对科学发展观等重大理论、实施的重大战略和驾驭经济社会发展所需知识的学习，优化知识结构，丰富知识储备，努力提高战略思维、创新思维能力和落实科学发展观的执行力。注重调查研究，对所做的工作要深入研究，用心把握，增强工作的预见性、系统性和创造性。在深入研究、科学论证的基础上，及早搞好全市“十二五”规划的编制工作，为我市未来五年经济社会发展提供科学指导和依据。强化实践锻炼，在改革发展的主战场、维护稳定的第一线、服务群众的最前沿经受考验、砥砺品质、增长才干，不断提高解决实际问题和应对复杂局面的能力。创新政府工作方式，整合集约利用各类行政服务资源，降低客商和群众获取政府服务的成本。

　 要切实转变工作作风。各级政府工作人员要进一步强化责任意识，提升思想境界，奋发努力，永不懈怠，用高标准要求和衡量自己的工作，在比学赶超中不断争先进位。树立正确的政绩观、价值观，大力弘扬求真务实、真抓实干的工作作风，强化任务、责任落实，严格考核奖惩，切实把各项工作落到实处。坚持廉洁从政、从严治政，加快推进惩治和预防腐败体系建设，严格落实廉政建设责任制，强化对财政资金和重大投资项目的审计监督。政府工作人员特别是领导干部要警钟长鸣，防微杜渐，自觉遵守廉洁自律各项规定，清清白白做人，干干净净做事。自觉树立过紧日子的思想，坚决杜绝劳民伤财的形式主义和各种铺张浪费现象。牢固树立执政为民理念，深入基层，深入群众，体察民情民意，关心群众疾苦，真心实意为群众办实事、办好事，努力建设人民群众满意的政府。

　 各位代表！做好今年的工作，任务艰巨而繁重，责任重大而光荣。让我们更加紧密地团结在以胡锦涛同志为总书记的党中央周围，深入贯彻落实科学发展观，在中共泰安市委的坚强领导下，解放思想，锐意进取，迎难而上，扎实工作，推动经济社会又好又快发展，为加快“建设经济文化强市、打造国际旅游名城”进程而努力奋斗！
